# Nutrient limitation in Atlantic salmon rivers and streams: Causes, consequences, and management strategies

**DOI:** 10.1002/aqc.3811

**Published:** 2022-03-29

**Authors:** Fionn R. Bernthal, John D. Armstrong, Keith H. Nislow, Neil B. Metcalfe

**Affiliations:** ^1^ Institute of Biodiversity Animal Health and Comparative Medicine University of Glasgow Glasgow UK; ^2^ Marine Scotland – Science Freshwater Fisheries Laboratory Faskally Pitlochry UK; ^3^ USDA Forest Service Northern Research Station Amherst Massachusetts USA

**Keywords:** catchment, catchment management, fish, habitat management, invertebrates, nutrient enrichment, nutrients, oligotrophic, stream

## Abstract

1. Freshwater catchments can experience nutrient deficits that result in reduced primary and secondary productivity. The most commonly limiting nutrients are nitrogen and phosphorus, either separately or together. This review considers the impact of increasing nutrient limitation in temperate basin stream and river systems, focusing on upland areas that currently or previously supported wild Atlantic salmon ( *Salmo salar* ) populations.
2. Anthropogenic changes to land use and increases in river barriers have altered upland nutrient dynamics, with particular impacts on salmon and other migratory fish species which may be net importers of nutrients to upland streams. Declining salmon populations may further reduce nutrient sources, reducing ecosystem and fisheries productivity below desired levels.
3. Experimental manipulations of nutrient levels have examined the impacts of this cultural oligotrophication. There is evidence that growth and biomass of juvenile salmon can be increased via appropriate additions of nutrients, offering potential as a conservation tool. However, further research is required to understand the long‐term effects of these additions on salmon populations and stream ecosystems, and to assess the vulnerability of downstream habitats to eutrophication as a result.
4. Although purposeful nutrient addition with the aim of enhancing and conserving salmonid populations may be justified in some cases, it should be undertaken in an adaptive management framework. In addition, nutrient addition should be linked to nutrient retention and processing, and integrated into large‐scale habitat restoration and recovery efforts.
5. Both the scientific and the management community should recognize that the ecological costs and benefits associated with adding nutrients to salmon streams may change in a non‐stationary world.

## 1 INTRODUCTION

Life depends on adequate supplies of key elements, such as carbon, nitrogen and phosphorus (Xia et al., 2018). These can shape the productivity of entire ecosystems, and their relative supply is widely recognized to have profound consequences at an ecosystem level. Aquatic systems may be particularly vulnerable to variation in nutrient supply, triggering a variety of ecological consequences with implications for conservation. High nutrient levels, often as a result of human influences, may result in eutrophication, which is characterized by changes to community structure through excessive growth of planktonic algae and periphyton (Page et al., 2012). Macrophyte growth can also increase, leading to the competitive exclusion of less nutrient‐tolerant species in affected water bodies (Bergheim & Hesthagen, 1990). Increased epiphytic algal growth on macrophytes may lead to a reduction in light availability, exacerbating the change in community composition from macrophyte dominated to algal dominance (Hilton et al., 2006; O'Hare et al., 2018). Further impacts of eutrophication include declines in dissolved oxygen, which can lead to sudden fish mortality especially if coinciding with warmer temperatures (Schinegger et al., 2016).

**FIGURE 1:**
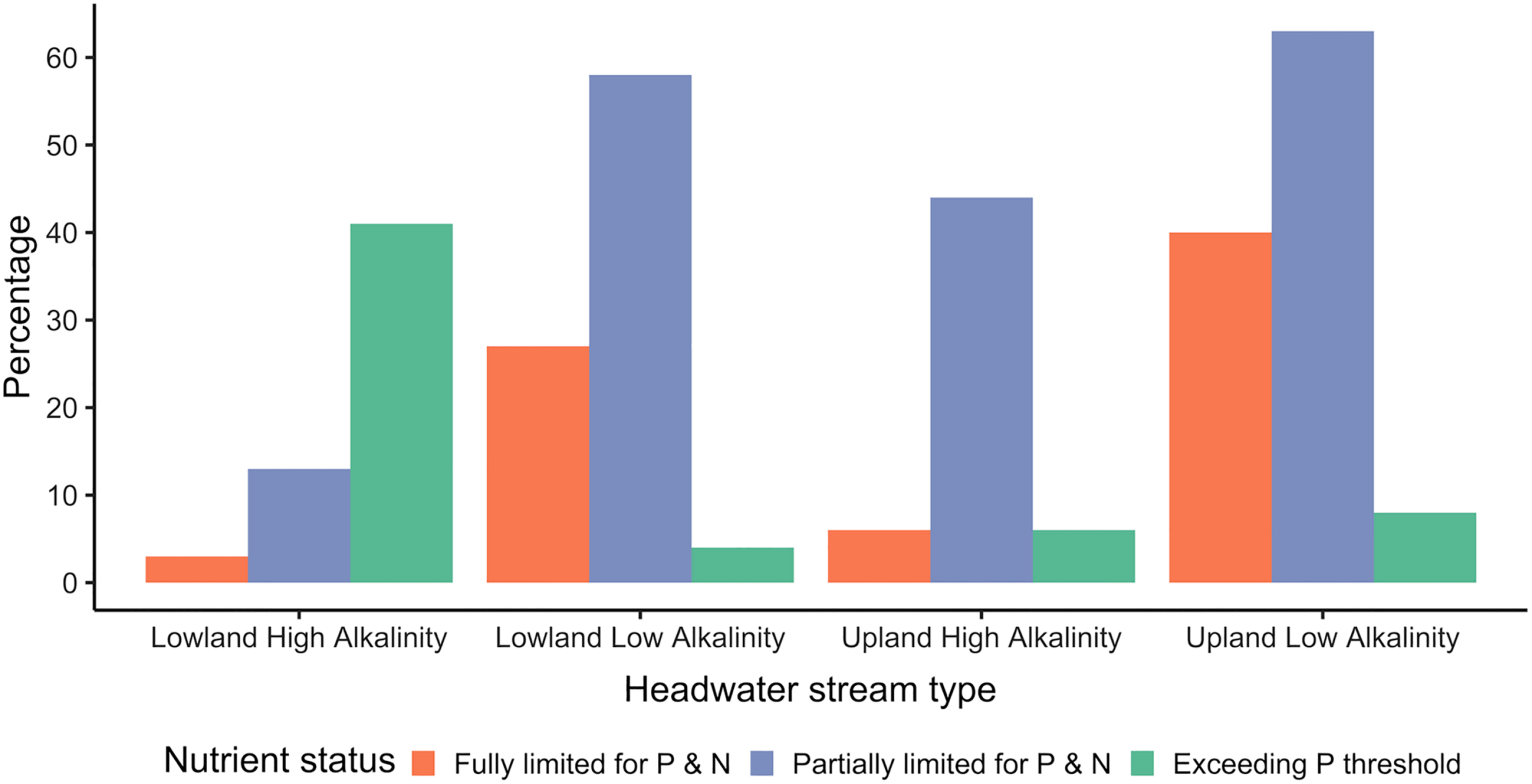
Levels of phosphorus and nitrogen in headwater streams in Great Britain in relation to elevation and alkalinity. Streams ‘exceeding P threshold’ show phosphorus concentrations that exceed 0.05 mg P L^−1^, and so are at risk of eutrophication; ‘partially limited’ streams are those in which P and N are moderately low, and ‘fully limited’ streams are those where P and N are so low as to cause significant limitation of primary productivity. A median elevation of 200 m separates ‘lowland’ and ‘upland’, and the boundary between ‘low’ and ‘high’ alkalinity is a mean alkalinity of 50 mg CaCO_3_ L^−1^. Adapted from data in Jarvie et al. (2018)

However, whereas eutrophication is more likely to be a feature of lowland systems, upland streams may be more likely to experience the other extreme of oligotrophication, where the biological demand for nutrients outstrips supply (Hecky & Kilham, 1988; Elser et al., 2007; Jarvie et al., 2018). As these upland streams can be tributaries of lowland rivers, eutrophication and oligotrophication can exist simultaneously at different locations within the same catchment (Figure 1; Stockner, Rydin & Hyenstrand, 2000). Upland streams are widely recognized as conduits that connect terrestrial and aquatic systems and influence downstream waters (Alexander et al., 2007). They are strongly influenced by runoff from surrounding hill slopes, and so receive sediments, biological matter and nutrients (Gomi, Sidle & Richardson, 2002). Despite these inputs, upland streams may experience nutrient limitation; usually a single element is lacking (typically P or more rarely N), or there can be co‐limitation when both P and N are scarce (Jarvie et al., 2018; Myrstener et al., 2018). Nutrient limitation reduces primary production by taking the availability of the key elements C, N and P away from the optimal ratio of 106C:16N:1P, termed the Redfield ratio (Redfield, 1958), with major impacts on the productivity and diversity of aquatic ecosystems (Smith, Jarvie & Bowes, 2017). Naturally low nutrient concentrations in upland streams can be reduced still further as a result of human activity (e.g. through habitat and land‐use change), a process called cultural oligotrophication (Stockner, Rydin & Hyenstrand, 2000).

The oligotrophic nature of upland streams may be partially offset by resource subsidies that cross ecosystem boundaries, often through the process of animal migrations (Doughty et al., 2016). Perhaps the most famous of these migrations acting as resource subsidies are the spawning migrations of salmon, both Pacific (*Oncorhynchus* spp.) and Atlantic ( *Salmo salar* L). Salmon spawn in fresh water, mostly in fast‐flowing tributary streams (Jonsson & Jonsson, 2011; Quinn, 2018). Juveniles (parr) spend a variable period of time (depending on the species) growing in fresh water before transforming into the seawater‐tolerant smolt stage and migrating to sea (Mobley et al., 2021). They gain weight rapidly at sea before returning to their natal stream to spawn (Quinn, 2018; Mobley et al., 2021). Their migrations from the oceans to the spawning grounds involve the transfer of large quantities of nutrients in the form of eggs, excreta and carcasses of spent adults, a process that is well documented in species of Pacific salmon (Gende et al., 2002; Schindler et al., 2003). Although the populations of spawning migrants (and hence the nutrients transferred) tend nowadays to be on a larger scale in species of Pacific compared with Atlantic salmon, there is evidence that Atlantic salmon populations were once far larger, even before the declines documented over the last century (Lenders et al., 2016) so that their baseline ‘natural’ population size (and hence level of nutrient transfer) is unclear. Nonetheless, even current populations of Atlantic salmon are capable of delivering significant levels of marine‐derived nutrients to tributary streams, with positive impacts on algal growth, invertebrate populations and juvenile fish growth (Nislow, Armstrong & McKelvey, 2004; McLennan et al., 2019).

**FIGURE 2:**
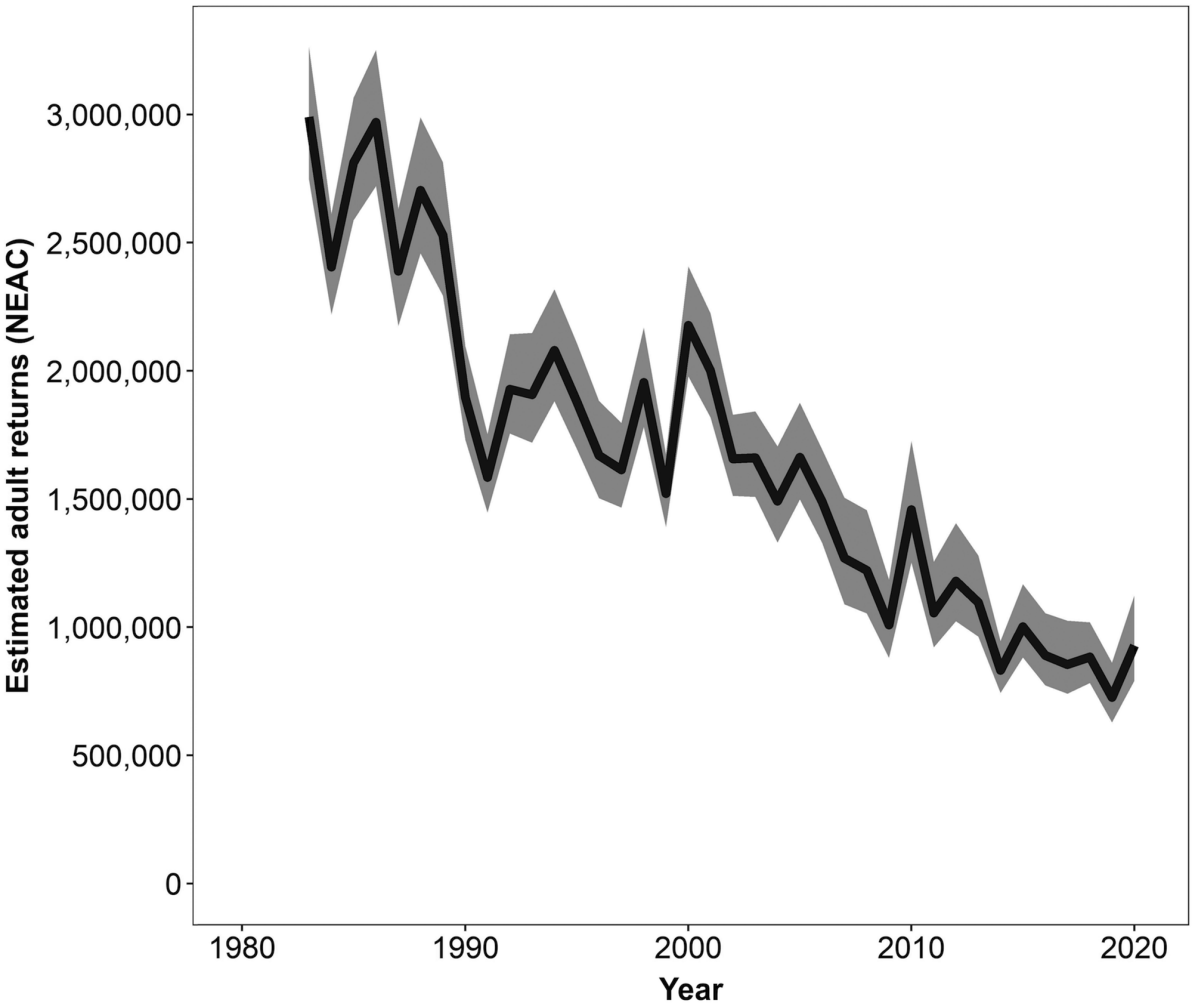
Variation over years in estimated numbers of Atlantic salmon returning to rivers within the North‐East Atlantic Commission (NEAC) (ICES Scientific Reports, 2021); 90% confidence bands shown in grey

The documented decline in populations of Atlantic salmon over recent decades (Figure 2) has occurred across much of their natural range (Chaput, 2012). Pressures on salmon are various, and operate in both the freshwater and marine environments (Beaugrand & Reid, 2012; Todd et al., 2012; Forseth et al., 2017; Olmos et al., 2020). These population declines are of serious concern, given the economic, cultural and conservation value of Atlantic salmon: in 2017, total expenditure from recreational angling alone was estimated to be €300–500 million across the North Atlantic (Myrvold et al., 2019). This has led to wide‐ranging conservation initiatives. For example, in the European Union, Atlantic salmon are designated for protection in freshwater habitats under Annexes II and V of the European Habitats Directive (Council of the European Communities, 1992). Under Annex II, core areas of habitat are required to be protected under the Natura 2000 Network, whereas for Annex V, member states are obliged to ensure that any exploitation in the wild is consistent with maintenance of a favourable conservation status.

With the closure of many commercial fisheries and control of recreational angling, conservation efforts have moved towards improving juvenile salmon survival and growth (and hence the production of smolts) through freshwater habitat restoration (Thorstad et al., 2021). This includes consideration of the impact of declines in resource subsidy in upland streams resulting from decreased spawner abundance. Lower nutrient inputs from spawners results in reduced growth rates of juvenile salmon (Auer et al., 2018; McLennan et al., 2019), and potential alterations to marine survival arising from changes in size attained by the time of smolt migration, as this is correlated with return rates (Armstrong et al., 2018; Gregory, Armstrong & Britton, 2018). This has led to the suggestion that nutrient restoration in spawning streams that have experienced cultural oligotrophication could be used as a conservation tool to manage and enhance important fish populations. However, cultural oligotrophication often escapes recognition in the literature as a key stressor limiting effective restoration efforts (Lennox et al., 2021). Such action could be part of a strategy to mitigate losses of salmon at sea to counter current declines in Atlantic salmon. However, this requires assessment of associated risks to receiving bodies of water and the scale of potential benefits. This is therefore a complex and potentially contentious issue that presents challenges for managers, practitioners, regulators and policy makers.

This review describes the impact of nutrient limitation in upland temperate streams (which are the typical spawning habitat of salmon) – a topic that has received far less attention than the issue of eutrophication further downstream. Evidence is presented that experimental nutrient additions to upland streams can increase stream invertebrate populations and the growth rates and biomass of the fish that feed on them. Given this complexity, our objective is to synthesise the state of science on nutrient limitation of aquatic ecosystem production, from the perspective of management of Atlantic salmon and the ecosystems within their current and historical catchments, but within a wider context of other anadromous freshwater fishes. The review is particularly oriented towards Holarctic river basins where migratory fishes are an important resource and play key roles in ecosystems. The aim is to inform conservation and restoration practice by providing an integrated perspective allowing policy makers and practitioners to identify relevant principles and case studies, as well as signposting areas of study warranting further attention.

## 2 SOURCES OF NUTRIENTS IN HEADWATER STREAMS

Nitrogen is supplied to headwaters mainly through atmospheric distribution, often originating from agricultural use and the combustion of fossil fuels, returning to land or water through wet and dry deposition (Boyer et al., 2006). It is abundant in the atmosphere but in an inert form (N_2_ gas), which must be transformed into reactive nitrogen to be biologically available (Stein & Klotz, 2016). As a consequence, in both Europe and North America a greater percentage of the total continental N inputs are of human origin (61% and 59%, respectively) than from natural sources such as nitrogen fixation (Boyer et al., 2006). In aquatic systems, organic nitrogen is degraded through ammonification producing ammonium and ammonia (NH_4_^+^, NH_3_) which then undergo nitrification leading to oxidation into nitrate (NO_3_) (Xia et al., 2018).

Phosphorus may be present in several different forms within a system. In natural waters it is usually present as inorganic phosphate (PO_4_^3−^), also known as orthophosphate, which may be present in either dissolved or particulate form, with particulate forms making up the majority of the P load (Spivakov, Maryutina & Muntau, 1999). As there are multiple P species, P can be measured in a variety of ways, usually involving the separation of particulate and dissolved P by filtration, after which separate measurements are made of the different fractions (Spivakov, Maryutina & Muntau, 1999). Soluble reactive phosphorus (SRP) is a measure of the dissolved inorganic P, usually orthophosphate, that is biologically available to plants and algae within a sample. The combined amount of all forms of P in a sample is defined as the total phosphorus (TP). Phosphorus may also be present in an organic form (i.e. bound to plant or animal tissue). Measurements of stream water P may not reflect the true amount of P within a system because organic P can also be taken up and used by algae (Whitton & Neal, 2011; Schoffelen et al., 2018). In addition, low concentrations of P in stream water may not always indicate limitation for primary production, as luxury uptake by algae during periods of high P availability can allow growth during periods of P scarcity, and thus may not appear in soluble P sampling (Jarvie et al., 2013). The sources of P are more complex than those for N, and so will be considered in more detail in the following sections.

### 2.1 Geological and atmospheric sources of phosphorus

Bedrock, soils and stream‐bed sediments are primary sources of P in upland streams (Bol et al., 2016), and parent lithology is a principal determinant of overall stream structure and function. Porder & Ramachandran (2013) showed that the concentration of P can vary 30‐fold among rock types, with the highest P concentrations found in iron‐rich, silica‐poor igneous rocks such as basalt. Sedimentary rocks may also be rich in P, with the highest concentrations in mudstone, claystone and siltstone, with P concentration reducing as grain size increases. Metamorphic rocks show broadly similar P concentrations to the rocks from which they are derived. Soil P availability is positively correlated with the P concentration of the underlying bedrock (Porder & Ramachandran, 2013), and this effect of bedrock can translate into SRP levels in the streams that run over them through the erosion of bankside soils and sediments (van der Perk et al., 2006). The presence of alkaline elements in these rocks increases P availability, so that more acidic streams are more likely to be P limited than where the alkalinity is high (Jarvie et al., 2018).

The P content of stream banks is determined in part by localized land use, but also from the deposition of upstream sediments (Fox, Purvis & Penn, 2016). Phosphorus, which has no gaseous phase, may also be supplied to catchments by atmospheric deposition as dust (Gibson, Wu & Pinkerton, 1995; Mladenov et al., 2012). Atmospheric P can be supplied in sufficient amounts to cause ecological effects in areas where the bedrock is nutrient‐poor (Vicars, Sickman & Ziemann, 2010). However, atmospheric deposition associated with early industrialization in lowland and coastal urban centres tended to acidify upland surface waters and reduce P availability, except in the most remote regions (Jüttner et al., 2021).

### 2.2 Biological sources of phosphorus

The input of material in the form of logs, sticks and leaves may exceed 1 kg m^−2^ year^−1^ in streams with heavily forested riparian zones; leaves form the dominant nutrient input owing to their quantity and rate of breakdown (Webster et al., 1999). A perhaps surprising P input to streams comes from pollen, which is high in phosphorus (Lee, Kenkel & Booth, 1996). Although the quantity of P supplied via pollen may be low, deposition in summer when biological demand is high has important implications for the overall P budget: indeed, in the Precambrian Shield catchment, Ontario, Canada, pollen accounted for up to 30% of TP deposition (Eimers, Hillis & Watmough, 2018). Most of the North Atlantic basin was originally forested and these forests have been subjected to major changes, including large‐scale deforestation. The replacement of native forests with plantation monocultures (primarily conifers) that are intensively managed for timber, alongside reductions in age‐class and species diversity, has probably resulted in reductions in the quantity of leaf and pollen inputs of P.

The movement and migration of animals results in the transfer of nutrients across ecosystem boundaries. As mentioned earlier, the spawning migrations of salmon and other anadromous fish species results in the release of gametes, excreta and (in some cases) carcasses of spent adults on or close to the spawning grounds. This often results in a net import of marine‐derived nutrients to upland systems (Gresh, Lichatowich & Schoonmaker, 2000; Gende et al., 2002; Schindler et al., 2003). The examples that have received the most attention to date are those associated with Pacific salmon. These are large‐scale migrations occurring across much of the Pacific Northwest, with up to 280 million salmon from five species of the genus *Oncorhynchus* migrating upstream every year, importing large quantities of marine‐derived nutrients such as C, N and P, but also smaller quantities of essential micronutrients such as calcium, iron, magnesium, sodium and cobalt (Gresh, Lichatowich & Schoonmaker, 2000; Schindler et al., 2003; Currier et al., 2020). These nutrient inputs support a wide variety of predators and scavengers, including bears, wolves, eagles, corvids and many other large vertebrates (Shardlow & Hyatt, 2013). However, the spawning behaviour of Pacific salmon can also lead to the export of nutrients from streams, often as a result of bioturbation from the excavation of nests in the stream bed. Pacific salmon spawning behaviour has been shown to shift ecosystems from primary production to heterotrophic production, and also export large quantities of nutrients downstream through increased transport of suspended sediment (Moore et al., 2007; Holtgrieve & Schindler, 2011).

Nutrient deposition is not limited to semelparous Pacific salmonids. The Atlantic salmon is an iteroparous species capable of repeat spawning, but many individuals may still die on or adjacent to the spawning grounds (Williams et al., 2010). The species was estimated to import 1.7–5.3 t of P each year to the River Tweed in northern England (Lyle & Elliott, 1998), and even in a short river in south‐west Norway the annual import from Atlantic salmon was 132 kg P (Jonsson, Jonsson & Hansen, 2003). The phenomenon also occurs in other anadromous species of the North Atlantic basin, such as the semelparous sea lamprey ( *Petromyzon marinus* ) (Nislow & Kynard, 2009; Weaver et al., 2015), the European river lamprey ( *Lampetra fluviatilis* ) (Masters et al., 2006) and several species of river herring (alosids) such as the alewife ( *Alosa pseudoharengus* ) (Barber et al., 2018). Although many adults of iteroparous species such as alewives and Atlantic salmon will return to the ocean after spawning, they nonetheless still excrete waste products (including P) while in fresh water. Moreover, P can be deposited in reproductive material, such as gametes or the mortality of embryos and fry. For instance, although alewives spawn in lakes, the streams through which they migrate are the recipients of their waste products, estimated to be 2.17 μg P per g of wet fish mass per hour (Post & Walters, 2009; West et al., 2010).

Fish spending the entirety of their lives in fresh water are also capable of playing a role in the transport of P. In North America, longnose suckers ( *Catostomus catostomus* ) migrate from the Great Lakes into tributary streams, with spawning populations reaching 10^2^–10^4^ individuals in small streams (Klingler, Adams & Heinrich, 2003). The proportion of suckers that die in the spawning streams is low, but the contribution from excretory products and eggs can be significant (Childress & Mcintyre, 2015). One difference between these nutrient sources is their availability: P in excretory products is more immediately available to primary producers than P contained in eggs, which requires mineralization in order to be taken up (Childress & Mcintyre, 2015; Childress & McIntyre, 2016). However, eggs are immediately available for consumption by stream‐resident fish (Childress & McIntyre, 2016). Other species of fish such as European and American eels ( *Anguilla anguilla* and *Anguilla rostrata* , respectively) may export nutrients from freshwater to marine systems, although this nutrient export has not been quantified.

### 2.3 Anthropogenic sources of nutrients

Anthropogenic inputs are increasingly important sources of nutrients in freshwater ecosystems but tend to be less significant in headwater streams than further downstream. In upland catchments, these may be grouped into atmospheric sources, point sources (e.g. wastewater discharge, such as from sewage treatment plants or sewer outflows), which tend to have a continuous flow, or diffuse sources (such as agricultural and urban runoff, septic tank leakage, logging, and construction) which are often interrupted and irregular (Carpenter et al., 1998). These sources of nutrients can be sufficient to cause changes to community structure. For example, P‐rich discharge from a wastewater treatment plant into an Austrian stream was shown to result in an 80% increase in mean daily macroinvertebrate secondary production further downstream, owing to an increase in the proportion of gatherers and grazer/gatherers (Singer & Battin, 2007). Withers et al. (2009) concluded that a large proportion of the anthropogenic inputs of nutrients into fresh waters may not be from agricultural fertilizers (as is commonly assumed), but from multiple diffuse sources in rural areas (see Withers & Jarvie, 2008 for review). For example, up to 25% of P in waste water originates from household detergents (Richards et al., 2015).

## 3 FACTORS CAUSING NUTRIENT LIMITATION

Although the streams in which salmon spawn receive nutrient inputs from multiple sources, these may be insufficient to prevent the habitats being oligotrophic. Before human influence, this limitation was primarily restricted to acidic catchments with naturally low nutrient levels; this form of oligotrophication does not require any remediation. Over more recent times, however, anthropogenic causes have become of overriding importance in some systems, leading to the phenomenon of cultural oligotrophication (Stockner, Rydin & Hyenstrand, 2000). The concept of nutrient limitation originates from Liebig's ‘Law of the Minimum’, with the ‘minimum’ being the nutrient present in the smallest proportion relative to the growth demands of an organism (Liebig, 1842; Harpole et al., 2011). Nutrient limitation is complex, with systems able to experience limitation by a primary nutrient, secondary limitation from another nutrient, or co‐limitation from two or more nutrients (Tank & Dodds, 2003). In aquatic systems, phosphorus and nitrogen are usually assumed to be the major limiting nutrients (Dodds & Welch, 2000). Phosphorus can become limiting when the N:P ratio exceeds 16:1, whereas N becomes the main limiting nutrient at lower N:P ratios (Redfield, 1958; Allan & Castillo, 2007).

The most extensive limitation in catchment streams is often found for P in upland low‐alkalinity areas, with more than 60% of such streams in Great Britain being partially limited for P and 40% fully limited; co‐limitation of P and N is also extensive (Jarvie et al. (2018); Figure 1). However, nitrogen is increasingly being recognized as a limiting nutrient in its own right (Jarvie et al., 2018). There is particular evidence for N limitation across boreal Fennoscandia, resulting in constraints on biofilm primary production; activities such as clear‐cutting result in the export of N downstream, contributing to further N losses (Burrows et al., 2015; Schelker et al., 2016). Another contributor to nitrogen limitation in upland streams is denitrification. During this process denitrifying microbes produce N_2_ gas from nitrates, which is lost to the atmosphere through the anaerobic respiration of nitrite (NO_2_^−^), nitric oxide (NO) and N_2_O, ultimately reducing the instream availability of nitrogen (Stein & Klotz, 2016). The percentage of nitrogen entering streams and rivers that is removed through this process varies among catchments, but has been estimated to be between 5% and 50% (Holmes et al., 1996; Galloway et al., 2004; Alexander et al., 2007).

### 3.1 Nutrient storage, retention and fate

Phosphorus and nitrogen may be stored in a variety of ways in upland catchments. On a small scale, microbes, algae, diatoms and cyanobacteria make up periphyton, forming biofilms on the substrate or on larger macrophytes. Periphyton can store significant concentrations of nutrients structurally within the polysaccharide matrix, and can also retain suspended particles (Battin et al., 2003; Godwin, Arthur & Carrick, 2009). Macrophytes, although less dominant than periphyton in upland streams, still play a role in storing P and N by buffering the water current and catching suspended material, varying seasonally with macrophyte growth (Riis et al., 2019). These processes may be further enhanced by epiphytic algae on the leaves of macrophytes, which take up P and N from the water column and may act to reduce water velocity, allowing further nutrient storage (O'Hare et al., 2018).

Downstream transport of P and N is closely linked to nutrient cycling. As nutrients are moved downstream, they may be cycled through different forms in a process known as ‘spiralling’ (Webster & Patten, 1979). During a single cycle of a spiral, a nutrient atom would pass through three compartments whilst being transported downstream: water, particulates and consumer phases, and the average distance over which this cycle is completed forms the ‘nutrient spiral length’ (Newbold et al., 1981). A short spiral or uptake length indicates a high biological demand, so in nutrient‐limited waters the uptake length would be expected to be low (Schade et al., 2011). Headwater streams are characterized by a low water volume to benthic area ratio, providing a greater capacity for exchange of P and N between inorganic and organic materials (Withers & Jarvie, 2008).

Land use changes can result in a reduced capacity for systems to both store and retain limiting nutrients. Over the past 150 years, the spread of low‐intensity agriculture in the North Atlantic basin (usually in the form of rough grazing) has led to some temperate upland stream catchments becoming P‐ and N‐export systems (Stockner, Rydin & Hyenstrand, 2000). Channelization (the widening, deepening and straightening of streams) is carried out as a means to improve land drainage and is widespread: in north‐west Europe, over one third of land is now drained for agriculture (Abbot & Leeds‐Harrison, 1998). This stream channel simplification leads to increases in water velocity, therefore reducing the potential for nutrient uptake (and incidentally increasing the risk of eutrophication further downstream as nutrients are less likely to be retained in the tributaries). Evidence for reduced nutrient retention in simplified channels comes from Austrian agricultural headwater streams, where average SRP uptake length was shortest in open meanders (0.5 km), followed by forested streams (1.9 km) and longer still in channelized reaches (3.8 km) (Weigelhofer, 2017).

Streams are hydrologically linked to wetlands and floodplains, which also provide nutrient storage and retention capacity. Wetlands are particularly effective at retaining N, being approximately twice as effective as lakes (Saunders & Kalff, 2001). Indeed, construction of artificial wetlands is used in the removal of nutrients from wastewater treatment plants, with uptake from plants playing a major role in N removal (Vymazal, 2007). In wetlands, nutrient storage by emergent macrophytes is particularly important since complex below‐ground structures assist in P and N storage and in trapping sediments. However, in the North Atlantic basin, these wetlands are under threat of being transformed to agricultural land or land for housing (Čížková et al., 2013).

The recent reintroductions of the North American and Eurasian beavers ( *Castor canadensis* , *Castor fiber* , respectively) in areas where these species have been extirpated may help to increase nutrient storage by altering hydrological regimes through dam construction, so creating ponds and wetlands. For example, Eurasian beavers reintroduced to headwater streams in eastern Scotland have been shown to reduce P and N concentrations by 46% and 43%, respectively, in water directly downstream of their dams compared with unmodified sites (Law, Mclean & Willby, 2016). However, the dams may prevent or impede fish migration, particularly under low‐flow conditions, while also increasing siltation, thereby reducing the availability of fish spawning habitat (Kemp et al., 2012).

The majority of nutrient transport (especially that of particulates) occurs during periods of peak flow (Martin & Harrison, 2011). Meyer & Likens (1979) demonstrated that within a stream in New Hampshire, USA, 46% of the annual P transport occurred in the short periods of time (less than 10%) when discharges were highest, although the concentration of dissolved P did not change with stream discharge. Sediment particle size also plays a role in nutrient cycling in upland streams (Gottselig et al., 2017). Phosphorus is transported 2–5 times further in particulate form than in the dissolved form, and fine particulates are readily colonized by bacteria (Froelich, 1988; Walters et al., 2014). Reductions in tree cover may increase soil erosion and sediment mobility, which, when combined with increased overland flow during rain events, may temporarily increase nutrient supply to streams, with deforested areas receiving greater pulses of particulates (Prairie & Kalff, 1988, but see Sweeney et al., 2004). Riparian buffer zones have previously been shown to reduce TP and N concentrations in streams, with wider buffers being more effective (Mayer et al., 2007).

Sediment and biological material transported during periods of high flows can enter lakes and reservoirs, and may accumulate in bed sediment, storing nutrients over long periods (Busteed et al., 2009). Human population growth, especially in the North Atlantic basin, has led to the construction of reservoirs and impoundments, which may lead to increased numbers of nutrient sinks in uplands. These may increase as hydropower gains in importance with the transition away from fossil fuels (Zarfl et al., 2015).

### 3.2 Reductions in nutrient inputs

Inputs of nutrients to upland streams can also be affected by human interventions, for instance through changes to forest composition or management. The removal of riparian vegetation, by reducing leaf litter inputs, may reduce a key source of nutrients (Webster et al., 1990). In general, rural uplands have steadily become depopulated as settlement, industry and agriculture have moved to the lowlands and coasts. Improvements in the efficacy of P removal from wastewater over time is also likely to have resulted in reduced P inputs. These reductions in anthropogenic sources of nutrients in upland streams may have contributed to P and N (co‐) limitation – a process that might continue even in the face of increasing global human populations.

The capacity for migratory fish to deliver P and N to upland streams is affected by the erection of impassable instream barriers – a process that in Europe has occurred over many centuries (Lenders et al., 2016). There are currently at least 1.2 million instream barriers on European rivers, with a mean density of one every 0.7 km (Belletti et al., 2020). Indeed, Duarte et al. (2021) showed that over half of European river networks have impaired connectivity for diadromous fish. In the USA, there are more than 80,000 dams and barriers reducing upstream connectivity, and this number does not include smaller, historical barriers (Magilligan et al., 2016). Although many weirs and dams now have incorporated structures that purportedly allow the passage of fish, some have limited effectiveness, letting through less than half the migratory fish biomass when compared with free‐flowing rivers (Noonan, Grant & Jackson, 2012). In recent years, however, conservation initiatives across Europe and the USA have led to the removal of river barriers, increasing upstream connectivity for migratory species including Atlantic salmon (Bellmore et al., 2019; Birnie‐Gauvin et al., 2020), and hence the potential for increased upstream nutrient transport.

The widespread decline in migratory fish populations (van Puijenbroek et al., 2019) has led to a reduction in P inputs to the headwaters. Gresh, Lichatowich & Schoonmaker (2000) report that in the Pacific Northwest USA, large declines in Pacific salmon populations mean that only 6–7% of marine‐derived P and N now reach inland waters compared with historical levels. Indeed, Moore et al. (2011) demonstrated that a shift from P import to P export occurred when spawning populations in Californian coastal streams decreased in size. Hence, recommendations have been made to set escapement targets for Pacific salmon at levels sufficient not just for egg deposition, but also to account for the return of adequate amounts of marine‐derived nutrients (Bilby et al., 2001), although it is unclear whether these recommendations have had any effect. The pattern of nutrient export is not limited to Pacific salmonids, as a net export of P was also demonstrated for Atlantic salmon when spawning populations declined (Nislow, Armstrong & McKelvey, 2004). Moreover, salmon stocked into upland streams as part of a mitigation response can cause sustained nutrient export contrary to the net nutrient importation by wild salmon when a system is unimpeded (Nislow, Armstrong & McKelvey, 2004).

## 4 CONSEQUENCES OF NUTRIENT LIMITATION FOR UPLAND RIVER SYSTEMS

Upland catchments are often remote, with little agricultural or urban nutrient inputs. Evidence that nutrients are often limiting in upland tributary streams comes from nutrient supplementation experiments that typically result in enhanced primary and/or secondary biomass (Peckarsky et al., 2013; Samways et al., 2015). Increases in the productivity of food webs can arise through alteration of biogeochemical cycling once systems are released from P and N limitation (Brailsford et al., 2019). There may also be changes to community composition through alterations in the proportion of different functional feeding guilds. For example, Demi et al. (2020) demonstrated a 52% increase in total organic‐matter flows to primary consumers in streams treated with aqueous P and N. Macroinvertebrates in this detritus‐based system were observed to reduce consumption of animal prey, but this was counteracted by an increase in the biomass of larger shredders. This system was also shown to be highly limited in P, with an increase of just 7 μg L^−1^ SRP being sufficient to significantly alter resource nutrient content (Demi et al., 2020).

Although an increase in nutrient availability is often shown to have the greatest impact at the base of food webs, the stimulation to autotrophic production can have cascading effects to the highest trophic levels (Bumpers et al., 2017), making it relevant in the context of fisheries management. These effects can arise through natural causes, as when the P inputs arising from alpine woodland wildfires led to increased algal and macroinvertebrate biomass, resulting in an increase in the size and weight of cutthroat trout (*Onchorhynchus clarki*) (Silins et al., 2014); however, of greater current interest is the concept of deliberate manipulation of nutrient levels.

## 5 NUTRIENT ADDITION AS REMEDIATION FOR CULTURAL OLIGOTROPHICATION

Adding nutrients to oligotrophic streams has been shown to have effects that propagate through the food web to higher trophic levels; for example, increasing the mean weight of under‐yearling salmonids of a range of species (Johnston et al., 1990; Slavik et al., 2004). Such observations have led to the concept of adding salmonid carcasses as a method of nutrient remediation for streams experiencing declining fish populations. These carcasses increase the immediate supply of nutrients such as SRP, often with a short‐term spike peaking after 2 weeks and then declining (Wipfli et al., 2010). The effect can be seen through invertebrate consumption of enriched biofilm, which is in turn taken up by fish. Another pathway is through direct consumption of carcass material by invertebrates and fish, as shown by Bilby, Fransen & Bisson (1996). Carcasses may also lead to increases in fish density (Bilby et al., 1998). Although experiments on carcass addition were initially focused on Pacific salmon, a growing body of literature has investigated the impacts that nutrient additions may have on juvenile Atlantic salmon (Table 1). It is clear that the addition of salmon carcasses or alternative nutrient sources has demonstrable effects at multiple levels within a food web, ultimately appearing to stimulate growth and biomass of juvenile Atlantic salmon, suggesting that increasing nutrient availability can have beneficial impacts on salmonid populations (Williams et al., 2009; Guyette et al., 2014; Auer et al., 2018; McLennan et al., 2019).

**TABLE 1:** Summary of impacts resulting from restoration of nutrients (in the form of adult salmon carcasses, carcass analogues or other marine‐derived nutrients (MDN)) to Atlantic salmon spawning areas in upland streams

Nutrient addition	Location	Study duration	Response variables	Result	Citation
Salmon carcasses	Scotland	4 months	Juvenile salmon biomass	Increase in juvenile salmon density, size and biomass	Williams et al., 2009
Salmon carcasses	Scotland	7 months	Carcass decomposition and invertebrate colonization	No detectable increase in stream water total P and N, rapid colonization by range of invertebrate taxa	Nislow et al., 2010
			Isotopic enrichment	δ^15^N enriched in periphyton, macroinvertebrate and juvenile salmon after carcass addition	
			Invertebrate abundance	Increased downstream of carcass sites	
Carcass analogue pellets mimicking June lamprey spawning and October salmon spawning	Maine, USA	2 years	Water chemistry	Increases in total dissolved P for 1 month	Guyette, Loftin & Zydlewski, 2013; Guyette et al., 2014
			Juvenile Atlantic salmon	Increases in mass and length in juvenile salmon	
			Atlantic salmon lipids	Treatment and temporal effects on total lipid	
			Isotopic enrichment	Higher in macroinvertebrates and juvenile Atlantic salmon	
MDNs from range of anadromous spawning fish	New Brunswick and Nova Scotia, Canada	10 months	Biofilm communities	Algal, fungal and bacterial abundance increased post‐MDN enrichment, positive effect on community standing stock, greatest in bacteria	Samways et al., 2015
			Biofilm δ^15^N enrichment	Significant during spawning, later returning to baseline levels	
MDNs from range of spawning anadromous fish	New Brunswick and Nova Scotia	7 months	Isotopic enrichment	δ^15^N and δ C enrichment in biofilm, macroinvertebrates and resident salmonids	Samways, Soto & Cunjak, 2018
			Reliance on MDNs	Parr derived 23% of nutrients from MDN spawning subsidies	
Carcass analogue pellets	Scotland	2 years	Macroinvertebrate biomass and abundance	Increases in nutrient‐treated streams	Auer et al., 2018; McLennan et al., 2019; Auer et al., 2020; McLennan et al., 2021
			Juvenile Atlantic salmon	Increases in length, body mass, biomass, but not density	
			Salmon natural selection	No longer selection for larger eggs or higher metabolic rate, and increased genetic diversity	
			Salmon standard metabolic rate	Higher standard metabolic rate individuals found in better microhabitats in control but not in nutrient‐treated streams	
			Salmon telomere length	Reduced rate of cellular ageing in poor microhabitats	
Salmon carcasses	Scotland	5 months	Atlantic salmon	Increase in juvenile survival but no impact on growth rates	Burton et al., 2020

The impact of nutrient additions is not limited to Atlantic salmon, having been demonstrated across a range of systems and taxa (Table 2). Periphyton and fish assemblages have been noted to change in response to slight increases in nutrients (Taylor et al., 2014). For example, P levels in upland streams have been linked to increased fish diversity: Gavioli et al. (2019) observed that higher P levels in Italian mountain streams were associated with an increased local contribution to overall diversity from native fish. In a Spanish headwater stream, N and P enrichment over 1 year resulted in changes to diatom community composition, with some species declining in abundance while others became more abundant, and some species were unaffected (Veraart et al., 2008). Changes in the trophic state of a water body, from oligotrophic to mesotrophic, may result in changes to invertebrate functional groups, which may have implications for larger ecosystem processes. For example, the biomass of shredders in stream leaf litter declined as the trophic level of streams increased from oligotrophic to hypertrophic in a French stream system (Baldy et al., 2007). Whereas studies have shown increases in macroinvertebrate abundance and biomass as a result of nutrient additions in the context of a conservation tool for Atlantic salmon (McLennan et al., 2019), the effect on macroinvertebrate diversity and functional groups is not yet known, and there are potential changes in ecosystem functioning that may only emerge after prolonged nutrient addition.

**TABLE 2:** Examples of experiments exploring the impact of adding phosphorus or other nutrients to upland temperate streams. For an extended summary, see Gerwing & Plate (2019)

Nutrient addition	Location	Study duration	Response variables	Result	Citation
Phosphorus (as liquid H_3_PO_4_)	Alaska, USA	16 years	Primary producers	Increase in standing stock and bryophyte coverage	Slavik et al., 2004
			Macroinvertebrates	Increased densities of some invertebrate taxa	
			Arctic grayling ( *Thymallus arcticus* )	Increased weight and growth rate	
Phosphorus (as liquid H_3_PO_4_), nitrogen (as liquid NH_4_NO_3_)	North Carolina, USA	2 years	Prey quantity, prey size and prey biomass of salamanders	Increase in prey size and number but not biomass, change in dietary composition compared with pre‐treatment	Bumpers et al., 2017
Sockeye salmon ( *Oncorhynchus nerka* ) carcasses	Alaska, USA	20 years	Stream‐bank tree growth	Increase in growth rate	Quinn et al., 2018
			Isotopic enrichment	Higher δ^15^N in needles	
Chum salmon ( *Oncorhynchus keta* ) carcasses	British Columbia, Canada	2 years	Juvenile coho salmon growth	Increased growth rate when fish were initially small and at high densities	Giannico & Hinch, 2007
			Pre‐smolt size	Increased size in some situations	
Coho salmon ( *Oncorhynchus kisutch* ) carcasses	Washington, USA	8 months	Population density	Increased juvenile salmonid densities	Bilby et al., 1998
			Body condition	Increase body condition of juvenile salmonids	
			Stomach contents	Evidence of feeding on eggs and carcasses	
Sea lamprey carcasses + key nutrients	Maine, USA	7 weeks	Chlorophyll *a*	Change in nutrient levels over time	Weaver, Coghlan & Zydlewski, 2016
			Macroinvertebrate isotopes	Enrichment in δ^13^C in some taxa	
Salmon carcasses and carcass analogues	Idaho, USA	4 years	Biofilm standing crop	Chlorophyll *a* and ash‐free dry mass (ADFM) increased for up to 6 weeks	Marcarelli, Baxter & Wipfli, 2014
			Phosphorus	Short‐term increase in soluble reactive P, total P and total dissolved P	
Salmon carcass analogue pellets	Idaho, USA	2 years	Periphyton chlorophyll *a* and AFDM	Increase in chlorophyll *a* and AFDM	Kohler, Rugenski & Taki, 2008
			Macroinvertebrate biomass	Increase in biomass but not density except in some taxa	
			Water chemistry	No detectable effect	

The use of carcasses may often not be practicable, which has led to the development of salmon carcass analogues, usually derived from salmon carcasses or other fishmeal and produced as dry pellets, with an N:P ratio of 6:1 (Pearsons, Roley & Johnson, 2007). These analogues contain a similar mixture of elements as carcasses, including P, N and C, although the rate of release is likely to differ due to their homogeneous composition. An alternative is to use bags of feed pellets produced by the aquaculture industry, which have traditionally been based on marine fishmeal. These analogues are widely viewed as having almost the same nutritional value as salmon carcasses themselves, and have been found to have broadly similar effects within streams, but limited removal to the riparian zone, in contrast to the transport of real carcasses by scavengers (Collins et al., 2015). Ease of storage and application has led to such carcass analogues becoming a common form of nutrient supplementation. Like real carcasses, they produce large increases in nutrient concentrations soon after being applied to a stream. Guyette et al., (2014) demonstrated a 4‐fold increase in P concentrations in treated versus untreated streams, with dissolved P levels elevated for up to 5 weeks. This elevation tends to lead to an increased abundance of benthic macroinvertebrates that form the majority of the diet of juvenile stream‐living fish. McLennan et al. (2019) demonstrated in Scottish streams that carcass analogues enhanced the growth of juvenile Atlantic salmon, concurrent with an increased abundance of macroinvertebrates. Similar results were obtained by Guyette, Loftin & Zydlewski (2013) in streams in Maine, USA. Increases in fish biomass in response to the addition of nutrient subsidies may thus be caused by faster growth rates of individual fish rather than changes in fish density (Collins et al., 2016; Auer et al., 2018; McLennan et al., 2019). Interestingly, Auer et al. (2018) showed higher Atlantic salmon genetic diversity in streams treated with carcass analogues, as a result of more salmon families having surviving representatives. The effects of nutrient additions are not always clear, however. Some studies have shown only limited effects of carcass analogues on stream communities, although they did increase SRP concentrations (Wipfli et al., 2010). In addition, the provision of carcasses and carcass analogues cannot fully replicate the effect of salmon spawning, as it omits the excretion of waste products and deposition of gametes as well as the bioturbation occurring during nest construction, so that the input and transport of nutrients is reduced.

## 6 APPLYING SCIENCE TO CONSERVATION AND MANAGEMENT

Management and conservation strategies for declining populations of Atlantic salmon often focus on the freshwater phase of the life cycle, where interventions are more easily facilitated than during the marine phase, and where the species is subject to domestic legal protection. Increasing both the number and quality of migrating smolts is recognized as a priority conservation strategy for the fish, both to combat low levels of marine survival but also to mitigate the impacts of environmental change (Thorstad et al., 2021). One way in which this might prove possible is to restore nutrient levels in culturally oligotrophic tributary streams in which they spend the first year or more of life, as the evidence presented above shows that nutrient limitation may be widespread in these streams and that nutrient restoration may result in faster growth of the fish and larger size‐at‐age (Guyette, Loftin & Zydlewski, 2013; Auer et al., 2018; McLennan et al., 2019). Size and condition (weight per unit length) of salmon smolts is directly correlated with subsequent marine survival (Armstrong et al., 2018; Gregory, Armstrong & Britton, 2018). Therefore, if the increased size of salmon parr that has been observed after nutrient additions results in larger smolts, there would be clear expected benefits in terms of numbers of returning adult salmon. Modelling by Benjamin et al. (2020) has demonstrated the potential for this method with chinook salmon (*Oncorhynchus tshawytscha*), with increases in potential smolt output and size. However, in some cases faster growth may result in salmon reaching the size that triggers smolting a year earlier, at a smaller smolt size (McLennan et al., 2019). In such cases, nutrient additions may result in lower *per capita* chances of survival at sea, but increased numbers within a cohort surviving to become smolts, because of less time in the river and reduced inter‐cohort competition. An additional factor is that faster growth may result in a greater proportion of male salmon maturing precociously as parr (Aubin‐Horth et al., 2006), which may have an effect on their chances of surviving to become smolts. Therefore, an increase in the size‐at‐age of juvenile Atlantic salmon will not necessarily translate into more or larger adult fish; the overall effect of nutrient restoration on numbers and sizes of anadromous salmon thus depends on how these demographic factors balance out, and so warrants future investigation (Table 3).

**TABLE 3:** Suggestions for future research regarding the potential use of nutrient restoration to support migratory fish populations (in particular, Atlantic salmon)

Knowledge gap	Issue	Relevant studies
*Geographical range*	Literature currently biased towards North America; no studies relevant to migratory salmonids at the southern edge of European range, where populations are most fragile	Almodóvar et al., 2019
*Taxonomic skew*	Existing literature too focused on *Oncorhynchus* salmon, which tend to transport nutrients on a scale very atypical for migratory fish. Information needed on iteroparous species and those spawning at lower densities	Guyette, Loftin & Zydlewski, 2013; Auer et al., 2018
*Method of adding nutrients*	More information is needed on how the method, dose and frequency of application of nutrients can be made most cost‐effective and environmentally sustainable	Pearsons, Roley & Johnson, 2007; Wipfli et al., 2010
*Lack of long‐term studies*	There is a need for multi‐year dosing experiments in order to understand long‐term effects on target species	Slavik et al., 2004
*Impact on rest of the catchment*	Little is known of the ‘safe’ level of nutrients that can be added to upland streams without causing eutrophication further downstream	
*Co‐limiting factors*	P is commonly viewed as the main limiting nutrient, but N and P may often be co‐limiting, other factors such as light levels may also constrain primary production	Jarvie et al., 2018
*Life‐history considerations*	Complex interactions among the growth rates, migration and mortality of fish influence both the direction and strength of nutrient transport, with potential feedbacks to fish vital rates and population sizes	McLennan et al., 2019
*Environmental change*	Expected increases in the frequency and magnitude of extreme flows will affect nutrient retention in streams, while increased temperatures will affect ectotherm energy budgets and nutritional requirements	Jonsson & Jonsson, 2009; Kovach et al., 2016

The majority of experimental studies of nutrient addition for Atlantic salmon have been of short‐term duration (usually lasting a year at most), hence the impact on salmon smolt and returning adult size and survival has not been assessed (Table 1). No study, including in other salmonids, has yet attempted repeated annual nutrient additions following a cohort of fish from hatching to returning spawners (Table 2). Clearly there is a need for longer‐term repeated dose experiments, especially since both empirical and modelling studies provide evidence that effects of nutrient addition tend to fade quickly once additions cease (Ericksen et al., 2009; Benjamin et al., 2020). However, these experiments are extremely challenging to design and undertake at an appropriate scale and level of replication (Table 4). Therefore, predictive modelling using the best available information on salmon demographics in response to growth variation based on short‐term experiments (Auer et al., 2018) is also recommended. Short‐term experiments also, by definition, are not examining streams in the state that may develop after years of nutrient supplementation, which is likely to be most relevant to applied management scenarios. For example, it may take some years for invertebrate communities to stabilize when nutrient levels are increased. Furthermore, the nutrient intervention may change the shape of the consumer pyramid (Leroux & Loreau, 2015) such that a greater biomass of salmon parr may ultimately support a larger predator population rather than increase the output of smolts. To overcome these problems, it may be possible to use extensive monitoring of invertebrate and juvenile salmon population responses to nutrients, coupled with water chemistry information, to build predictive models of the changes in production that could be achieved through nutrient restoration. Advantages and limitations of these experimental and observational approaches are summarized in Table 4.

**TABLE 4:** Comparison of advantages and limitations of observational, small‐scale experimental and large‐scale adaptive management approaches to assessing effects of nutrient status on salmon populations

Approach	Advantages	Limitations
*Observational*	Large quantities of empirical data can be collected using natural variations across landscapes in real‐world situations and interrogated with multivariate modelling. The scenario may be immediately highly relevant to potential outcomes of changing nutrient state under prevailing environmental conditions.	Power to detect effects of any one variable likely to be limited especially (i) at extremes of variable distributions, which is often the case for low nutrients, and (ii) where there are interactions among habitat variables. The distributions of variable distributions are not controlled and therefore are likely to be unbalanced.
*Small‐scale experimental*	Tight control enables high power to detect effects of small changes in nutrient levels on a number of response variables (e.g. salmon number, size, condition, probability of early smolting or maturity).	Challenging logistics, usually relatively short‐term and limited to a specific set of general habitat conditions. Consequently, results may not be generalizable to multiple real‐world situations.
*Adaptive management and monitoring*	Facilitates rapid application of nutrient additions in real‐world management scenarios based on best available information, while checking for potential damage and assessing potential benefits. The approach potentially provides large temporal and spatial scale and possibility of monitoring at various life stages.	Challenging to organize replication needed to provide power to detect effects of nutrient interventions with confidence. Substantial resource is required to sustain high‐quality monitoring efforts across potentially multi‐generational timespans.

It is important to recognize that Atlantic salmon are also vulnerable to environmental changes as a result of a changing climate (Thorstad et al., 2021). This intersects with nutrient dynamics along several dimensions. Warmer and wetter conditions are predicted as a result of climate change, with increased heavy rainfall (Alexander et al., 2006). The predicted greater frequency and intensity of extreme precipitation and associated flood flows has important implications both for upland rivers and lowland receiving waters. Phosphorus and nitrogen inputs to streams and rivers may therefore increase over the short term because of an increased frequency and magnitude of floods. However, these nutrients may be rapidly lost in the uplands as a result of increased rates of transport from flood flows, while further downstream, the receiving waters will experience higher nutrient loading rates and greater risk of eutrophication. The balance between these processes is complex, but there is a clear need to manage riparian and floodplain habitats to hold back water and so retain nutrients in the upper reaches of catchments as much as possible.

As fish are ectothermic, a rise in water temperature will result in greater metabolic costs. In the high‐latitude cold water aquatic ecosystems that support salmonids, studies suggest that increasing water temperatures during the spring may result in the potential for increased salmonid growth and larger body size, but only if the food supply is not limiting (Bacon et al., 2005; Xu, Letcher & Nislow, 2010; O'Gorman et al., 2016). Deliberate nutrient addition could therefore mitigate some adverse effects of climate change by maintaining sufficient prey availability and supporting growth and production as streams warm. However, a further complication is that warmer downstream receiving waters may be more vulnerable to oligotrophication (Arora, Tockner & Venohr, 2016; Bolotov et al., 2018).

It should always be borne in mind that the addition of nutrients to streams that may be of important conservation value is not without contention. Manipulating nutrient levels in oligotrophic streams that may be considered to have high ‘naturalness’ (Boon et al., 2002) requires assessment of various trade‐offs and uncertainties in a rapidly changing world. Impacts on receiving waters and the surrounding habitats are important considerations, together with evaluation of whether such nutrient inputs might result in alterations to river or stream conservation or ecological status under legislation including the European Habitats Directive (Council of the European Communities, 1992) and the European Water Framework Directive (Council of the European Communities, 2000). In general, nutrient restoration may be suitable within catchments designated for their conservation value only if there would be no deleterious consequences for designated species, habitats, or other characteristics. Aiming to return to a historical baseline is widely agreed to be contentious and often not attainable in a non‐static world, but if there is evidence of a reduction in salmon abundance over previous decades then the restoration of nutrients might be considered a return to a more ‘natural’ state (sensu Boon et al., 2002), such as existed before human impacts. However, the addition of nutrients may have the potential to downgrade the ecological status of rivers. For example, under the Water Framework Directive, nutrient supplementation might cause a stream to lose its designation of ‘high ecological status’ (‘species composition and abundance correspond totally or nearly totally to undisturbed conditions’; Council of the European Communities, 2000) and instead be classified as having ‘good ecological status’ (‘slight changes in species composition and abundance from the type‐specific communities attributable to anthropogenic impacts on physicochemical and hydromorphological quality elements’). At present, the potential impacts of an adaptive nutrient remediation strategy on the conservation status of rivers are unknown, as the current research in this area cannot adequately answer these large‐scale uncertainties without further long‐term study.

## 7 CONCLUSIONS AND RECOMMENDATIONS

Consideration of stream water chemistry and land/water/fisheries management history suggest that P and N are likely to be limiting to juvenile fish production in temperate upland river systems, and that nutrient addition may increase production of juvenile salmon through a combination of increases in survival and individual growth rates. However, further understanding is required to determine how such responses vary among different river systems and community structures, how they may affect a stream's conservation value, and how these effects map on to changes in numbers and sizes of adult (including precocious male) salmon (Table 3).

Given these considerations, we feel that the stage is set for incorporating nutrient restoration into the management of salmonid fisheries in the region, but with some caveats. As an overarching concern, we propose that wherever possible, additions of P and N should be coupled with actions (such as restoration of habitat and channel complexity, increasing flow path length in channelized reaches, fostering floodplain–channel connectivity) that enhance the ability of upland systems to retain and process limiting nutrients while also increasing their naturalness. This will serve the dual purpose of allowing these nutrient additions to be more effective *in situ* and limiting negative downstream impacts; they will also have additional ecosystem and fish habitat benefits. Nutrient restoration can therefore be coupled with habitat management such as planting riparian trees to provide additional protection from climate change by shading and also enhancing local nutrient retention and cycling (O'Briain, Shephard & Coghlan, 2017).

Multiple replicates are required in appropriately balanced designs (Underwood, 1994) to measure the effects of nutrient additions. Potentially such experiments may incorporate paired comparisons between bifurcating tributaries to increase power to detect experimental manipulation of nutrients by controlling for other environmental variables (e.g. rainfall, geology and temperature) (Table 4). In view of the difficulty of conducting such large‐scale experiments, we recommend that an adaptive management approach is adopted. This approach would fast‐track likely benefits while providing the capacity to identify and minimize any damage due to inadvertent eutrophication. Such an approach will require the application of well coordinated and designed management and monitoring regimes. In addition, the use of linked ecosystem modelling approaches, such as the Aquatic Trophic Productivity model, coupled with salmonid life cycle models, may help to provide insights into the relationship between nutrient additions and habitat restoration efforts, as these have previously shown the potential benefits for salmonids through carcass restoration (Bellmore et al., 2017; Benjamin et al., 2020). These approaches may provide a framework for the results of these small‐scale but focused studies to contribute to more integrated answers.

In conclusion, nutrient restoration may well have the potential to help conserve and enhance protected Atlantic salmon populations in river systems that have experienced cultural oligotrophication. However, a combination of continued experiments and modelling, incorporating large‐scale adaptive management monitoring, is required to evaluate and refine the approach and minimize the risk of potentially adverse effects.

## CONFLICT OF INTEREST

The authors declare no conflict of interest.

## Data Availability

Data sharing is not applicable to this article as no datasets were generated or analysed during the review.
